# Bench, Bedside, Toolbox: T-Cells Deliver on Every Level

**DOI:** 10.3389/fimmu.2014.00031

**Published:** 2014-02-03

**Authors:** Bruno Laugel

**Affiliations:** ^1^Institute of Infection and Immunity, Cardiff University School of Medicine, Cardiff, UK

**Keywords:** T-cells, pMHC, TCR, cancer immunotherapy, immunosuppression

Decades of research have shed light on many aspects of T-cells, spawning a myriad of diagnostic and therapeutic applications in the process. Chief among those properties is the unique ability of T-cells to scan the intra-cellular protein content to detect anomalies in tissues, be it the presence of pathogens or cellular transformation. The tri-partite interaction between the T-cell receptor (TCR), its co-receptor CD4 or CD8, and peptide-major histocompatibility (pMHC) ligands determines the outcome of an encounter between a T-cell and an antigen presenting cell and can result in ignorance or trigger a cellular activation program central to adaptive immunity. Over the years, tremendous insights into the rules that govern this interaction have been gained at the molecular and cellular levels, resulting in the development of technologies and tools that improve our understanding of the dynamics of antigen-specific T-cell responses *in vivo* as well as therapeutic modalities aimed at harnessing the power of T-cells through vaccines, cellular therapies, and biologics. The selection of 18 articles that constitute this Research Topic reflects these advances in many ways and provides a snapshot of the current focus in the field, with an emphasis on the efforts made in order to translate our knowledge of T-cell biology into tools for therapy, diagnosis, and immune-monitoring.

From a fundamental point of view two primary research articles examine how T-cells discriminate between pMHC antigens and integrate signals that result in different cellular outcomes. Schaft et al. tested a panel of altered peptide ligands of human glycoprotein (gp)100 and identified a partial agonist that dissociates signaling networks downstream of TCR triggering (1). The altered peptide ligand they identified elicits cytotoxicity but negligible or no cytokine secretion nor NFAT-mediated transcription, an intriguing observation that appears related to the extent of binding by TCR and CD8α and reveals the intricacies of signal transduction downstream of the TCR. In an extensive study of T-cell activation, van den Berg et al. examined the response of a human CD8^+^ T-cell clone against several agonists of different affinities for the TCR (2). Their results support a model of epitope discrimination at the cellular level based on the integration of TCR signals, whereby the sum of signals read by a T-cell determines the functional response, rather than by the individual properties of receptor–ligand interactions. These two reports further highlight the analog nature of signal processing in T-cells, which enables diverse functional outcomes based on the concatenation of input signals rather than a binary response mediated via a simple on/off switch mechanism.

Also in the domain of basic research the articles by Li et al. and Szomolay et al. offer comprehensive insights into the roles of the co-receptors CD4 and CD8. In the former article, the authors summarize the literature on the structural and biophysical properties of the pMHC/co-receptor interaction and discuss the implications on the topological organization of the entire antigen receptor machinery on the T-cell membrane, a parameter that likely influences the initiation and transduction of TCR signals (3). Szomolay et al. focus on the modulation of antigen recognition and ligand specificity by the co-receptor CD8 (4). Based on existing experimental data they formulate mathematical models that predict dynamic variations of T-cell response specificity and magnitude as a function of pMHCI/CD8 binding kinetics and of CD8 expression levels on the cell surface, the latter phenomenon likely constituting an adaptive mechanism tuning responsiveness at different developmental stages. On the subject of antigen specificity, Wooldridge describes in details the extent of the cross-reactivity inherent to the TCR and the consequent degeneracy of T-cell antigen recognition (5). These parameters have clear implications when it comes to the pre-clinical development of T-cell based therapies, especially with respect to safety issues that relate to potential off-target effects.

Moving closer to translational research Burrows and Miles discuss the different parameters to consider when selecting TCRs for use in cellular therapy or as biologics (6). Again this article emphasizes the importance of assessing the antigen specificity and degeneracy profiles of therapeutic TCR candidates both in syngeneic and allogeneic systems. On the flip side of the TCR/pMHC interaction, Pentier et al. propose strategies to optimize T-cell epitopes in the context of therapeutic vaccination, including the design of synthetic antigen mimics that could circumvent the labile nature of native l-amino-acid peptides (7). Also relevant to the optimization of peptide ligands, Holland et al. provide fascinating insights into peculiar- and little-appreciated aspects of MHC class II epitope presentation, namely the influence of flanking residues that extend outside the MHC groove, on the interaction between the TCR and its antigen as well as T-cell activation (8).

A remarkable technological advance of molecular immunology has been the use of recombinant pMHC molecules to monitor T-cell responses by flow cytometry. Schmidt et al. review the development of these tools in detail from their initial description as monomeric reagents used to probe T-cell clones by photo-affinity labeling to their popularization as tetramers and higher order multimers for accurate and detailed *ex vivo* analysis of polyclonal T-cell responses (9). The authors also give an extensive account of recent technical improvements made in the manufacture of “switchable” class I pMHC multimers for the isolation of “untouched” antigen-specific T-cells and class II pMHC molecules and the challenges inherent to antigen-specific analysis of CD4+ T-cell responses by flow cytometry. As further illustration of the great strides made in pMHC technology Evavold and colleagues summarize the groundbreaking 2-dimension adhesion frequency assay they have developed and that allows monitoring TCR/pMHC interactions in their natural membrane environment (10). They also define new ways this technology can be used to advance our understanding of T-cell biology, for instance the detection and characterization of elusive CD4+ T-cells.

A large part of the Research Topic focuses on T-cell based cellular cancer therapies, perhaps the most promising domain of therapeutic application of T-cell biology at the moment. This approach has seen recent remarkable clinical success and is currently actively pursued around the globe. Kerkar starts by giving a general overview of T-cell based therapies for cancer and other disease indications (11). In addition to classical T-cell re-direction using viral vectors expressing TCRs or chimeric antigen receptors (CARs) the author discusses different therapeutic strategies using T-cells as vehicles such as the delivery of cytokines to diseased tissues. Kunert et al. remind us of the recent clinical successes of T-cell adoptive therapies by offering a comparative overview of clinical trials evaluating different experimental therapies in development, including immune checkpoint blocking antibodies and small molecule inhibitors (12). The authors proceed to define what parameters likely determine the success rates of TCR gene therapy, from the choice of target antigens to the cues that influence T-cell fitness or pre-conditioning patient treatment, and suggest strategies to overcome current challenges in the field.

Since the vast majority of tumor-associated antigens are directly derived from self proteins most naturally occurring peripheral TCRs bind to tumor pMHC with low affinity compared to microbial epitopes. Consequently, antigen receptor engineering that seeks to optimize and improve the recognition of tumor epitopes by increasing the affinity of the TCR is an important focus in the field of cancer cellular therapies. Stone and Kranz review in detail the TCR affinity-optimization efforts to date, mostly based on *in vitro* protein evolution platforms such as yeast and phage display, highlighting the benefits of the approach in terms of enhanced anti-tumor reactivity but also its pitfalls, in particular risks of autoimmune adverse effects in the case of high-affinity TCRs cross-reacting with non-tumor self epitopes (13). The authors further suggest strategies to identify potential off-target cross-reactive epitopes during the pre-clinical development of affinity-optimized TCRs. On the same topic Zoete et al. argue in favor of a rational, structure-guided approach to TCR/pMHC affinity-optimization (14). The authors describe their *modus operandi* to this endeavor, which is based on the *in silico* modeling of mutations within the complementary determining region loops of the TCR based on solved and modeled structures of TCR/pMHC complexes. An important take home message of these articles is that affinity enhancement should be within the physiological range of affinities observed for natural TCRs as supra-normal affinities seem to both result in inefficient activation as well as enhanced cross-reactivity. However, with respect to cross-reactivity, this view is somewhat counterbalanced by the article of Cole et al. who report the first structure of a high-affinity TCR generated by random mutagenesis and isolated by phage display (15). This TCR only bears mutations within the hypervariable CDR3β loop and owes its enhanced binding properties to additional contacts with the peptide rather than the MHC molecule, explaining the relative lack of increase in affinity for known cross-reactive ligands compared to the index epitope. Directed mutations that seek to mimic this design may be the way forward for TCR affinity-optimization.

Even though the articles of this topic focus heavily on the use of TCRs for cancer cellular therapy this shouldn’t play down the promises of CARs, which have also shown spectacular clinical results. This small injustice is repaired thanks to the article of Hombach and Abken, who review recent CAR engineering principles intended to promote long-term persistence and functionality of re-directed T-cells *in vivo* by triggering co-stimulatory signaling pathways subsequent to antigen engagement (16).

In addition to receptor engineering, a complementary and promising avenue to improve the efficacy of T-cell based cancer cellular therapies lies in the inactivation of immune-suppressive mediators of the tumor milieu. Recent clinical successes obtained with blocking antibodies targeting CTLA-4 or PD-1 as monotherapies raise the question of whether combining such approaches with T-cell adoptive transfer would provide additional clinical benefit, as it is hoped it will with vaccines. In accordance, Rufer and colleagues discuss TCR affinity-optimization along with other potential therapeutic strategies that include targeting co-inhibitory receptors with blocking monoclonal antibodies, impairing downstream inhibitory signaling and second messenger pathways with small molecule inhibitors or activating co-stimulatory receptors with agonistic antibodies (17). Generally speaking the combination of T-cell therapy with the inactivation of co-inhibitory receptors expressed by T-cells is a recurrent theme in the articles of the research topic and in the broader literature. The implementation of such therapeutic interventions is also a matter of discussion. Co-administration of blocking monoclonal antibodies or recombinant proteins with cellular therapies is usually the most popular option. However, recent progress in genome engineering technologies offers new angles for co-inhibitory receptor inactivation in the context of cellular therapies. Lloyd et al. briefly review the literature on protein-guided and RNA-guided endonucleases as a means to inactivate specific genes in human cells (18). They hypothesize that the co-delivery of anti-tumor antigen receptors with genome editing agents targeting immune checkpoint receptor genes may represent a cost-efficient and safe way of improving cancer ACTs without the need for combining different therapeutic modalities such as the adoptive transfer of cells as well as the infusion of biologics.

In summary, these 18 articles give an overview of several themes currently under investigation, and of their challenges, in the field of human T-cell biology. It is noteworthy that a large part of the Research Topic addresses applied aspects of T-cell immunology; this might be an indication that decades of intense fundamental research might be about to pay off and translate into effective treatments as well as viable commercial products.

## References

[1] Schaft N Coccoris M Drexhage J Knoop C De Vries IJ Adema GJ An altered gp100 peptide ligand with decreased binding by TCR and CD8alpha dissects T cell cytotoxicity from production of cytokines and activation of NFAT. Front Immunol (2013) 4:270 10.3389/fimmu.2013.00270 24027572 PMC3762364

[2] van den Berg HA Ladell K Miners K Laugel B Llewellyn-Lacey S Clement M Cellular-level versus receptor-level response threshold hierarchies in T-cell activation. Front Immunol (2013) 4:250 10.3389/fimmu.2013.00250 24046768 PMC3763380

[3] Li Y Yin Y Mariuzza RA Structural and biophysical insights into the role of CD4 and CD8 in T cell activation. Front Immunol (2013) 4:206 10.3389/fimmu.2013.00206 23885256 PMC3717711

[4] Szomolay B Williams T Wooldridge L Van Den Berg HA Co-receptor CD8-mediated modulation of T-cell receptor functional sensitivity and epitope recognition degeneracy. Front Immunol (2013) 4:329 10.3389/fimmu.2013.00329 24151493 PMC3801161

[5] Wooldridge L Individual MHCI-restricted T-cell receptors are characterized by a unique peptide recognition signature. Front Immunol (2013) 4:199 10.3389/fimmu.2013.00199 23888160 PMC3719040

[6] Burrows SR Miles JJ Immune parameters to consider when choosing T-cell receptors for therapy. Front Immunol (2013) 4:229 10.3389/fimmu.2013.00229 23935599 PMC3733007

[7] Pentier JM Sewell AK Miles JJ Advances in T-cell epitope engineering. Front Immunol (2013) 4:133 10.3389/fimmu.2013.00133 23761792 PMC3672776

[8] Holland CJ Cole DK Godkin A Re-directing CD4(+) T cell responses with the flanking residues of MHC class II-bound peptides: the core is not enough. Front Immunol (2013) 4:172 10.3389/fimmu.2013.00172 23847615 PMC3696884

[9] Schmidt J Dojcinovic D Guillaume P Luescher I Analysis, isolation, and activation of antigen-specific CD4(+) and CD8(+) T cells by soluble MHC-peptide complexes. Front Immunol (2013) 4:218 10.3389/fimmu.2013.00218 23908656 PMC3726995

[10] Blanchfield JL Shorter SK Evavold BD Monitoring the dynamics of T cell clonal diversity using recombinant peptide: MHC technology. Front Immunol (2013) 4:170 10.3389/fimmu.2013.00170 23840195 PMC3699728

[11] Kerkar SP “Model T” cells: a time-tested vehicle for gene therapy. Front Immunol (2013) 4:304 10.3389/fimmu.2013.00304 24098300 PMC3784795

[12] Kunert A Straetemans T Govers C Lamers C Mathijssen R Sleijfer S TCR-engineered T cells meet new challenges to treat solid tumors: choice of antigen, T cell fitness and sensitisation of tumor milieu. Front Immunol (2013) 4:363 10.3389/fimmu.2013.00363 24265631 PMC3821161

[13] Stone JD Kranz DM Role of T cell receptor affinity in the efficacy and specificity of adoptive T cell therapies. Front Immunol (2013) 4:244 10.3389/fimmu.2013.00244 23970885 PMC3748443

[14] Zoete V Irving M Ferber M Cuendet MA Michielin O Structure-based, rational design of T cell receptors. Front Immunol (2013) 4:268 10.3389/fimmu.2013.00268 24062738 PMC3770923

[15] Cole DK Sami M Scott DR Rizkallah PJ Borbulevych OY Todorov PT Increased peptide contacts govern high affinity binding of a modified TCR whilst maintaining a native pMHC docking mode. Front Immunol (2013) 4:168 10.3389/fimmu.2013.00168 23805144 PMC3693486

[16] Hombach AA Abken H Young T cells age during a redirected anti-tumor attack: chimeric antigen receptor-provided dual costimulation is half the battle. Front Immunol (2013) 4:135 10.3389/fimmu.2013.00135 23761793 PMC3672777

[17] Hebeisen M Oberle SG Presotto D Speiser DE Zehn D Rufer N Molecular insights for optimizing T cell receptor specificity against cancer. Front Immunol (2013) 4:154 10.3389/fimmu.2013.00154 23801991 PMC3685811

[18] Lloyd A Vickery ON Laugel B Beyond the antigen receptor: editing the genome of T-cells for cancer adoptive cellular therapies. Front Immunol (2013) 4:221 10.3389/fimmu.2013.00221 23935598 PMC3733021

